# Closing system-wide yield gaps to increase food production and mitigate GHGs among mixed crop–livestock smallholders in Sub-Saharan Africa

**DOI:** 10.1016/j.agsy.2015.12.006

**Published:** 2016-03

**Authors:** B. Henderson, C. Godde, D. Medina-Hidalgo, M. van Wijk, S. Silvestri, S. Douxchamps, E. Stephenson, B. Power, C. Rigolot, O. Cacho, M. Herrero

**Affiliations:** a Commonwealth Scientific and Industrial Research Organization, Queensland Bioscience Precinct, 306 Carmody Road, St Lucia, QLD 4067, Australia; b ILRI, International Livestock Research Institute, Nairobi, Kenya; c University of New England, Armidale, NSW 2351, Australia

**Keywords:** Productivity, Yield gap, Emission intensity

## Abstract

In this study we estimate yield gaps for mixed crop–livestock smallholder farmers in seven Sub-Saharan African sites covering six countries (Kenya, Tanzania, Uganda, Ethiopia, Senegal and Burkina Faso). We also assess their potential to increase food production and reduce the GHG emission intensity of their products, as a result of closing these yield gaps.

We use stochastic frontier analysis to construct separate production frontiers for each site, based on 2012 survey data prepared by the International Livestock Research Institute for the Climate Change, Agriculture and Food Security program. Instead of relying on theoretically optimal yields—a common approach in yield gap assessments—our yield gaps are based on observed differences in technical efficiency among farms within each site. Sizeable yield gaps were estimated to be present in all of the sites. Expressed as potential percentage increases in outputs, the average site-based yield gaps ranged from 28 to 167% for livestock products and from 16 to 209% for crop products. The emission intensities of both livestock and crop products registered substantial falls as a consequence of closing yield gaps.

The relationships between farm attributes and technical efficiency were also assessed to help inform policy makers about where best to target capacity building efforts. We found a strong and statistically significant relationship between market participation and performance across most sites. We also identified an efficiency dividend associated with the closer integration of crop and livestock enterprises. Overall, this study reveals that there are large yield gaps and that substantial benefits for food production and environmental performance are possible through closing these gaps, without the need for new technology.

## Highlights

• We estimate yield gaps for mixed crop–livestock smallholder farmers in seven sites.
• Yield gaps are based on differences in technical efficiency among farms.
• Yield gaps ranged from 28 to 167% and 16 to 209% for livestock and crop products.
• Closing these yield gaps results in substantial falls in product emission intensities.
• There is an efficiency dividend from crop and livestock integration.

## 1 Introduction

Smallholder farming systems in Sub-Saharan Africa are known to have sizeable yield gaps (Tittonel and Giller, 2013, Dzanku et al., 2015, Nin-Pratt et al., 2011) and to therefore have large potential for increasing food production. The yield gap concept is commonly used in agronomic assessments, which compare observed yields with maximum potential yields under certain agroecological conditions for a particular region. As noted by Neumann et al. (2010); Nin-Pratt et al. (2011), and Dzanku et al. (2014) these potential yields are often overestimated because they are based on optimal conditions (e.g. where pests and diseases are effectively controlled) and often ignore practical regional and farm-level constraints (Rockström and Falkenmark, 2000). A number of recent studies that focus on Africa and the globe, use statistical and mathematical programming approaches based on variations in observed yields, which can provide more realistic yield gap estimates (Neumann et al., 2010; Dzanku et al. 2014; Tittonel and Giller, 2013, Baldos and Hertel, 2012, Foley et al., 2011, Licker et al., 2010). These and other yield gap studies for Africa (Mutegi and Zingore, 2014) and the globe (Rockström and Falkenmark 2010) are, however, limited as they do not include livestock. This is a significant omission given that most food production in Sub-Saharan Africa comes from mixed crop–livestock systems (Herrero et al. 2010).

Variations in farm productivity arise because of differences in production environments, production technologies, and the efficiency of production processes (Lovell et al., 1994). The scope for closing yield gaps depends on the degree to which each of these factors is responsible for the gap. For instance, it may not be possible to close the portion of the yield gap that is caused by an unfavourable production environment, because environmental variables such as precipitation are, for the most part, not under the discretion of farm managers. Conversely, part of the yield gap can be closed through management decisions including more precise matching of agronomic inputs and crop requirements (technical efficiency improvement), and through the adoption of more productive technologies such as improved animal breeds (Nin-Pratt et al., 2011).

This study is concerned with improving yield gaps through improvements in the efficiency of production, and it is based on the construction of separate production frontiers for mixed crop–livestock smallholder farmers in seven Sub-Saharan African sites covering six countries (Kenya, Tanzania, Uganda, Ethiopia, Senegal and Burkina Faso). In response to the growing interest from the research community in exploring nexus between productivity and environmental performance (Burney et al., 2010, FAO, 2010, Gerber et al., 2013), we also assess the impacts of closing yield gaps on GHG emission intensities for livestock and crop products.

The frontier-based approach used in this study is also used by Neumann et al. (2010) and Baldos and Hertel (2012) to estimate global yield gaps for crops, however we consider both crops and livestock. The production frontiers estimated in this study are based on the most technically efficient farms within each site, and they represent the maximum amount of output that can be produced from the existing production inputs used by each farm. To accommodate the multiple-output nature of these production systems, we estimate distance functions using a stochastic frontier analysis (SFA) estimation procedure. This is a robust methodology with sound theoretical underpinnings in production economics, which also permits the statistical significance testing of model specifications (Coelli et al., 2005, Bogetoft and Otto, 2011).

## 2 Data

Given the importance of livestock production among smallholders in Sub-Saharan Africa, this study addresses a crucial gap in the literature by estimating system-wide yield gaps for in these production systems. We use the term “system-wide” to convey that all farm production inputs and outputs (inclusive of both crops and livestock) are included in our yield gap estimates. Previous studies have only assessed yield gaps within crop enterprises. It also makes a unique contribution by exploring the effects of yield improvements for multiple outputs on the emission intensity of production.

The data for farm production and farm attribute variables used in this study come from the IMPACTlite database prepared by International Livestock Research Institute for the Climate Change, Agriculture and Food Security programme (Rufino et al., 2013), and is based on farm household surveys conducted over the 2012 calendar year. This study focuses on seven of the nine Sub Saharan African sites in this database covering six countries. Five of the sites are in East Africa (Nyando and Wote (Kenya); Hoima (Uganda); Lushoto (Tanzania); and Borana (Ethiopia)) and two are in West Africa (Yatenga (Burkina Faso); and Kaffrine (Senegal)). The two sites in West Africa are situated at less than 350 m altitude and have annual rainfalls ranging from 400 to 800 mm, with substantial year to year variability (Table 1). The sites in Eastern Africa show strong spatial heterogeneity of climate and topography, with low annual rainfall in Wote and in Borana and much higher rainfall in Hoima and Lushoto. Furthermore, rainfall predictability in Eastern Africa is relatively high and helps to reduce risks of crop failure. The remoteness, measured by proximity to nearest city, varies among the sites, with Borana being relatively more remote than that other sites. The key challenges that both West and Eastern Africa are facing are an increasing population, water stress, widespread land erosion, declining soil fertility and high climate variability (Förch et al., 2013).

A selection of some the main production inputs and outputs from the surveys is also provided in Table 2. There is a diversity of production systems both across the sites, ranging from the agro-pastoral system in Borana, characterized by large ruminant herds relative to farm land area, to the more crop-based systems in Hoima and Kaffrine. Grain production is important in all sites, with vegetable production also significant in Hoima, Yatenga and Lushoto. Local ruminant breeds predominate across the study areas, with cross bred cattle more common in parts of Lushoto, Nyando (Rufino et al., 2013). The average farm sizes are similarly small in most sites with the exception of Hoima and particularly Kaffrine, where the farm sizes are appreciably larger.

From the survey data we constructed two aggregate output variables, one for livestock and one for crops, and five input variables including land, labour, animals, materials, and farm assets. All of the variables, except for land (ha) and labour (hours), are composites for which indices were required to aggregate their various components. For animals, tropical livestock unit (TLU) index was used to aggregate different animal types. This index takes into account the feed requirements of different animals and is therefore reflective of their varying resource requirements (ILRI (International Livestock Research Institute), 2011, FAO, 2003). The standard measure for one TLU is one cattle with a body weight of 250 kg. By contrast a 30 kg sheep or goat with is equal to 0.2 TLUs, and is therefore assumed to consume 20% as much feed as 250 kg cow. For farm assets we relied on ILRI (International Livestock Research Institute) (2011) and BMGF (Bill and Melinda Gates Foundation) (2010) to aggregate different asset classes. These included all productive farm assets including items such as ploughs, water pumps and wheelbarrows. The values assigned to each type of asset reflect their relative economic values. For example, a powered water pump has a value twelve times greater than a shovel and three times greater than a plough (ILRI (International Livestock Research Institute), 2011, BMGF (Bill and Melinda Gates Foundation), 2010). For livestock products, crop products and materials (which includes fertilizers, seeds, pesticides, herbicides, feeds, vaccinations) we constructed Fisher quantity indexes (Diewert, 1992). This required the use of quantity and price data from the IMPACTlite household survey database and, compared to most economic index numbers, the Fisher index has a number of desirable statistical properties including dimensional invariance (i.e. it is independent of the units of measurement used) and proportionality (i.e. if all quantities increase by the same proportion, then the index will increase by the same proportion) (Coelli et al., 2005). The Fisher indexes are dimensionless quantities that are relative to a “base farm” in each sample and, as such, they have no interpretative value and do not warrant inclusion in Table 2. For the purposes of exposition we have included Fertilizer, one of main components of the Material input. Similarly the main livestock and crop products are also displayed in Table 2. The Fertilizer input summarized in Table 2 is a composite of all synthetic fertilizers used in each site. We have included the exact selection of input and output variables, including the Fisher quantity indexes that were used in each stochastic frontier model, in the supplementary materials (Table S1).

After cleaning the data and removing incomplete records we were left with an average of 146 farms per site: 181 in Nyando (3 observations removed); 150 in Wote (21 observations removed); 147 in Hoima (2 observations removed); 145 in Lushoto (30 observations removed); 168 in Borana (22 observations removed); 127 in Yatenga (25 observations removed); and 101 in Kaffrine (25 observations removed). Incomplete records were those identified as those missing key production inputs (e.g. land, labour, farm assets) as well as those with input data, but and no reported outputs.

Seven farm and farmer attribute variables were also assembled and used in the analysis to test if they could explain some of the variations in farm performance in each site. These variables are displayed in Table 3 and they include: age of farm household head (years); gender of farm household head (dummy variable: value of 1 for female and 0 for male); off farm income (proportion of total household income from outside the farm); market participation (proportion of farm products that are sold, based on their value in local currency units); domestic assets (aggregated using index in ILRI (International Livestock Research Institute) (2011)) as a measure of overall household wealth; livestock specialization (proportion of total farm products that come from livestock, based on their value in local currency units); and household size (number of persons living in the farm household).

There are clear differences between the sites with regard to all of the attributes apart from the average ages of the farm household heads (Table 3). There are particularly large disparities in the reliance of off farm income, livestock specialization and market participation. The geographically remote site of Borana has the lowest share of income from off farm sources, the lowest proportion of farm products sold into markets, and the highest specialization in livestock production. This reflects the fact that ruminant production is possible on poor quality land, unsuitable for crop production, and often located in remote areas that are less accessible to markets. This relationship is supported by Frelat et al. (2016) who, using similar data sources, show that farm households that have poor market access are particularly dependent on livestock to meet their food energy requirements. At the other end of the spectrum is Hoima, with the highest degree of market participation and lowest reliance on livestock production.

## 3 Methods

### 3.1 Stochastic frontier analysis

Frontier efficiency methods for estimating technical efficiency have been around since mid-last century (following the work of Malmquist (1953); Shephard (1953) and Farrell (1957), who introduced the concepts of distance functions and efficiency measurement), but rose to prominence in the field of production economics much later (Coelli et al., 2005). The two main approaches for measuring technical efficiency are stochastic frontier analysis (SFA) and data envelopment analysis (DEA). These approaches are conceptually very similar however SFA uses econometric methods (Aigner et al., 1977, Meeusen and van den Broeck, 1977), while DEA relies on mathematical programming (Charnes et al., 1987). The strengths and weaknesses of each approach have been extensively discussed in the literature (Coelli et al., 2005, Bogetoft and Otto, 2011). The main difference is that DEA, being non-parametric, does not impose a functional form on the data and is therefore the more flexible of the two approaches. SFA, on the other hand, requires the selection of a functional form for the production frontier. While this reduces the flexibility of the approach, SFA can deal with statistical noise arising from measurement errors, data anomalies and uncertainties, and the incomplete specification of functions. This capacity to deal with statistical noise explains the term “stochastic” in SFA, and it is property which is particularly useful for the studies in developing countries where measurement and reporting errors are hard to avoid. With DEA, statistical noise from these factors will affect the position of the frontier and, consequently, technical efficiency scores. For this reason we selected the SFA approach to estimate separation production frontiers for each of the seven study sites.

The SFA frontier describes the maximum possible level of production given the amount of all production inputs used in the sample population, taking into account both statistical noise and technical inefficiency; the latter causing farms to lie below the frontier. As we are dealing with mixed farm systems that have multiple outputs, we use a multi-output distance function approach. We choose a transcendental logarithmic (translog) functional form due to its flexibility and other desirable properties such as the ability to impose homogeneity, and the concavity of the transformation function to the origin (Coelli and Perelman, 2000).

The translog distance function with M (*m* = *1*, *2*, …, *M*) outputs and K (*k* = *1*, *2*, …, *K*) inputs, and for I (*i* = *1*, *2*, …, *I*) firms, is given by:

(1) $$\ln\;D_{\mathrm{Oi}}=\alpha_{0}+\sum_{m}\alpha_{m}\;\ln y_{\mathrm{mi}}+\frac{1}{2}\sum_{m}\sum_{n}\alpha_{\mathrm{mn}}\ln y_{m}\ln y_{\mathrm{ni}}+\sum_{k}\beta_{k}\ln x_{\mathrm{ki}}+\frac{1}{2}\sum_{k}\sum_{l}\beta_{\mathrm{kl}}\ln x_{k}\ln x_{\mathrm{li}}+\sum_{k}\sum_{m}\gamma_{\mathrm{km}}\ln x_{\mathrm{ki}}\ln y_{\mathrm{mi}}$$

where “O” indicates an output-orientated distance function.

The restrictions required for homogeneity of degree one in outputs are $\sum_{m}\alpha_{m}=1$, $\sum_{n}\alpha_{\mathrm{mn}}=0$, $\sum_{m}\gamma_{\mathrm{mn}}=0$, while symmetry restrictions require *a*_*mn*_ = *a*_*nm*_ and *β*_*kl*_ = *β*_*lk*_. A convenient method of imposing the homogeneity constraint upon Eq. (1) is to follow Lovell et al. (1994) and arbitrarily choose one of the outputs, such as the *M*th output, to normalize the function:

(2) $$\ln\left(\frac{D_{\mathrm{Oi}}}{y_{1i}}\right)=\alpha_{0}+\sum_{m}\alpha_{m}\ln\left(\frac{y_{\mathrm{mi}}}{y_{1i}}\right)+\frac{1}{2}\sum_{m}\sum_{n}\alpha_{\mathrm{mn}}\ln\left(\frac{y_{\mathrm{mi}}}{y_{1i}}\right)\ln\left(\frac{y_{\mathrm{ni}}}{y_{1i}}\right)+\sum_{k}\beta_{k}\ln x_{\mathrm{ki}}+\frac{1}{2}\sum_{k}\sum_{l}\beta_{\mathrm{kl}}\ln x_{k}\ln x_{\mathrm{li}}+\sum_{k}\sum_{m}\gamma_{\mathrm{km}}\ln x_{\mathrm{ki}}\ln\left(\frac{y_{\mathrm{mi}}}{y_{1i}}\right)$$

Including both a symmetric random error term (for statistical noise) and an asymmetric error term (for inefficiency) into the model, requires the rewriting of the technical inefficiency measure ln*D*_*Oi*_ as −* u*_*i*_. The random error term *v*_*i*_ can then be added to the translog function, which is now redefined in terms of ln*y*_1*i*_ as:

(3) $$\ln y_{1i}=\alpha_{0}+\sum_{m}\alpha_{m}\ln\left(\frac{y_{\mathrm{mi}}}{y_{1i}}\right)+\frac{1}{2}\sum_{m}\sum_{n}\alpha_{\mathrm{mn}}\ln\left(\frac{y_{\mathrm{mi}}}{y_{1i}}\right)\ln\left(\frac{y_{\mathrm{ni}}}{y_{1i}}\right)+\sum_{k}\beta_{k}\ln x_{\mathrm{ki}}+\frac{1}{2}\sum_{k}\sum_{l}\beta_{\mathrm{kl}}\ln x_{k}\ln x_{\mathrm{li}}+\sum_{k}\sum_{m}\gamma_{\mathrm{km}}\ln x_{\mathrm{ki}}\ln\left(\frac{y_{\mathrm{mi}}}{y_{1i}}\right)+v_{i}+u_{i}$$

Following Paul et al. (2000) we transform the left side of the equation to be ln*y*_1*i*_ rather than − ln *y*_1*i*_. This causes the signs of the coefficient estimates corresponding to the distance function to be reversed, so that they conform to the expected signs of standard production function models, easing the interpretation of the results. We explore the impacts of farm attribute variables, such as farmer age and market participation, on inefficiency by including them as components of the *z*_*i*_ vector, where:

(4) $$u_{i}=\delta_{0}\sum_{j}\delta_{j}z_{\mathrm{ji}}$$

where the δ_j_s are unknown parameters to be estimated and *z*_*ji*_ (j = *1*, *2*, …, *J*) is a column vector of technical inefficiency explanatory variables. We used maximum-likelihood methods to estimate the stochastic translog distance function with the usual distributional assumptions for the *v*_*i*_ and *u*_*i*_ terms: the *v*_*i*_ are random variables assumed to be i.i.d. (independently and identically distributed) *N*(0, *σ*_*u*_^2^); and the *u*_*i*_ are nonnegative random variables independently distributed as truncations at zero of the *N*(*m*_*i*_, *σ*_*u*_^2^) distribution where *m*_*i*_ = *δ*_*j*_*z*_*ji*_. Estimation was carried out using the FRONTIER econometric package developed by Coelli and Henningsen (2014) for implementation in R software.

By using output distance functions, the technical efficiency scores estimated in this study quantify the maximum extent to which output can be produced from existing production inputs, and from existing practices and technologies.

### 3.2 Calculation of GHG emission intensities

Baseline GHG emissions from livestock (CH_4_ and N_2_O from animals and manure management) and crops (N_2_O emissions from fertilizers, manures and plant residues) were calculated using Tier 1 methods outlined in Intergovernmental Panel on Climate Change (IPCC) guidelines (IPCC, 2006). Emission intensities were then calculated by dividing the emissions from livestock and crop production by their corresponding products, expressed in terms of protein and energy equivalents, respectively. The various livestock outputs were converted into protein equivalents based on protein conversion factors from Opio et al. (2013) and MacLeod et al. (2013), and then aggregated into ruminant and poultry products. Similarly the crop products were converted into energy equivalents based on the USDA Food Composition Database (USDA, U.S. Department of Agriculture, 2015), and then aggregated into separate grains and beans/pulses products. Emission intensities were estimated for both the observed or baseline situation and a fully efficient scenario in which the yield gaps in each sample population are assumed to be closed. To estimate the emission intensities in the fully efficient scenario we first had to estimate the new output quantities associated with closing the yield gaps. Given that the technical efficiency scores provide a proportional measure (between 0 and 1) of actual output relative the maximum achievable output for each farm, this was achieved by simply dividing the observed output levels of each farm by its respective technical efficiency score. This is a valid approach because the technical efficiency values in this study are based on the simultaneous expansion of all outputs, while holding inputs fixed and maintaining the same proportional mix of outputs observed in the baseline. Since the closing of yield gaps is based on expanding outputs without changing baseline input levels, GHG emissions were treated the same as the production inputs, and left unchanged as a result of closing the yield gaps. Consequently, we assume that yield gaps are closed as a result of improved management of existing resources (e.g. better animal husbandry to increase the productive lifespan of animals and more precise matching of agronomic inputs to meet crop growth requirements) rather than through changes in practices and technologies (e.g. a switch to more energy rich feeds, heavier animal breeds, and higher agronomic input systems).

## 4 Results

### 4.1 Yield gaps and technical efficiency

The average technical efficiency score for smallholders at each site ranges between 0.43 and 0.72 (Table 4). The efficiency scores are also expressed as potential yield gaps by converting them to percentage increases in output for each site. This conversion was simply performed by calculating the percentage increase required to increase each aggregate efficiency score from their estimated value to a value of 1. This is a coarse measure of the yield gap, because it gives equal weight to each farm within each site and does not differentiate between products. More disaggregate yield gap estimates addressing each of these limitations are provided later in Table 5. The yield gaps range from a 39% increase in Kaffrine to more than doubling of output in Lushoto and Borana. These are encouraging findings, as they show there is scope to generate reasonably large increases in output with existing practices and existing levels of input use. The variance in yield gaps tends to be greater in sites with lower mean technical efficiency scores (e.g. Nyando and Borana), as shown by the coefficients of variation (CV) in Table 4. This is expected, because sites with a larger spread in performance should generally have larger yield gaps.

**Fig. 1:**
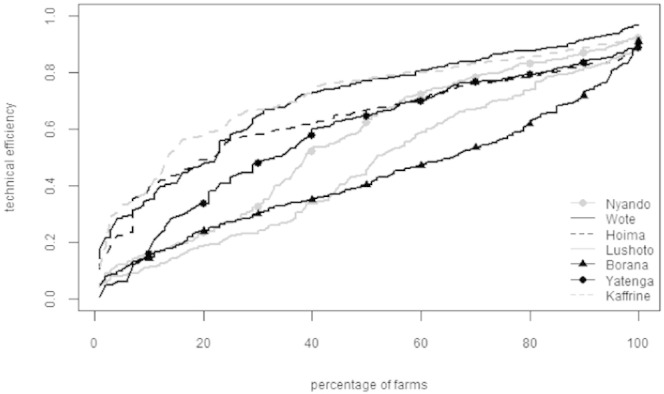
The distribution of farms by technical efficiency in each study site.

The distributions of the farm level technical efficiency scores, assembled in increasing order within each site, are presented in Fig. 1. As with the mean scores, these distributions vary quite widely between sites. A relatively large proportion of smallholders in Wote and Kaffrine, in particular, clustered around high efficiency scores in excess of 0.6. Whereas farmers in Borana, Lushoto and Nyando are spread much more uniformly across all efficiency levels, reflecting the findings of relatively large average yield gaps in these sites (Table 4). Market participation appears to have some attenuating influence on the size of yield gaps for some sites, as indicated by the high efficiency scores in the more market orientated Kaffrine and Hoima sites, and the relatively low scores in Borana. The statistical significance of the relationship between the farm attribute variables and efficiency within each site are reported later in this section. The complete list of parameter estimates of the stochastic frontier models for each site is also included in the supplementary material file associated with this paper.

To further explore the potential gains from closing yield gaps, we report output targets which could be achieved by closing yield gaps for a selection of the main livestock and crop products in each site (Table 5). As previously explained, the yield gap targets in Table 5 are calculated by dividing the observed farm outputs for each farm by its respective technical efficiency score. While the magnitude of these changes broadly corresponds to the mean yield gaps in Table 4, some differences emerge due to variations in product mixes and efficiency levels among farms. For instance, in Lushoto the potential for product expansion is generally higher than its average yield gap. This is because relatively more inefficient farms, with larger yield gaps, assume a greater output share of the main products in this site.

The results outlined above indicate that there is a wide range of farm performance within each site. Below we report on the inefficiency effects component of the estimated models, to see if the variations in performance can be explained by the farm attributes listed in Table 6. This table contains the coefficient values estimated for each farm attribute variable along with an indication of its level of significance. As the inefficiency effects in the models are a linear function of these observed variables (Battese, 1995), a negative sign on the coefficients in Table 6 indicates a negative relationship with inefficiency, or a positive relationship with technical efficiency.

The Market participation coefficient is negative in all sites and statistically significant in five of these. Thus the more market-orientated farmers that are able to sell a greater proportion of their outputs are more efficient. Similarly, domestic asset wealth has quite a consistent negative correlation with inefficiency, however this relationship is only significant in Yatenga. In contrast, farms that obtain a higher share of their income from off farm sources were consistently more inefficient, with this relationship being statistically significant in four sites. The link between livestock specialization and inefficiency is more mixed, although it tends to be positively related to inefficiency in the sites where the coefficient is statistically significant. Interestingly, there appears to be an efficiency dividend associated with the closer integration of crop and livestock enterprises. The four sites where livestock specialization is positively related to inefficiency are the most livestock dependent. Whereas this variable is negatively related to inefficiency in the sites that depend more on crops, with the only statistically significant and negative coefficient in Lushoto, the most crop-dependent site assessed.

A reasonably consistent picture emerges with regard to the age and gender. Age is positively related to inefficiency in most sites (although only statistically significant in Nyando and Hoima), suggesting that older farmers tend to be less efficient. Whereas Gender was positively related to inefficiency in the three sites for which this relationship was significant, suggesting that female headed farms face greater challenges regarding farm management than their male counterparts. The policy implications of these relationships between farm performance and farm characteristics are explored in the Discussion and conclusions section.

The ability to use statistical hypothesis tests in SFA is an important advantage for this method over non-parametric approaches such as DEA. In Table 7 we provide results from the null hypothesis that inefficiency effects are absent from the model, i.e. that all deviations from the frontier are the result of random noise instead of inefficiency. This hypothesis is rejected at the 5% level of significance or less in five of the seven sites, and at the 10% level of significance for two sites (Table 7).

We also report results of the null hypothesis that the inefficiency effects are not a function of the farm attribute variables in Table 8. This hypothesis is strongly rejected across all sites at between 0.1% and 1% levels of significance. Thus even though many of these attribute variables are individually insignificant, the joint effects of these variables are significant in each site.

### 4.2 Environmental impacts (GHG emission intensities)

As discussed, Improvements in technical efficiency can also deliver environmental benefits in terms of reducing the GHG emission intensity of farm products as well as improving natural resource use more generally. To illustrate this potential we calculate the emission intensities for livestock products (Table 9) and crop products (Table 10), before and after closing yield gaps. As expected, the emission intensities of poultry products are considerably lower than for ruminant products in every site. The baseline emission intensities within each livestock product class are of a similar order of magnitude, with the exception of poultry products in Kaffrine, which are higher owing to the focus on broiler rather than egg production; with the former being much less efficient (MacLeod et al., 2013). Significant reductions in the emission intensities of livestock products are possible across all sites, with falls of between 20 and 63% possible for ruminant products and between 27 and 61% for poultry products.

## 5 Discussion and conclusions

Despite the importance of livestock production in Sub-Saharan Africa, the issue of yield gaps among either livestock or mixed crop–livestock smallholders in this region is severely under-researched, as previous yield studies have focused on crops. This study addresses an important gap in the literature by estimating yield gaps for both crops and livestock in these production systems. Moreover, by considering the expansion of outputs for given levels of production inputs for each farm, the production improvements identified in this study can be assured of increasing total factor productivity without the risk of inadvertently making farmers economically worse off. By contrast, the improvement of partial productivity indicators (e.g. output per animal or per hectare) can result in greater use of inputs that are not considered in these indicators and thereby cause total factor productivity and farm profits to fall.

There are substantial yield gaps in the mixed smallholder farm communities assessed in this study, and closing gaps would provide marked benefits for smallholder incomes, food supply and environmental performance. We estimate that there is the potential to raise the production of the main livestock products from 28 to 167%, and the main crop products from 16 to 209%. There do not appear to be any clear regional patterns, as sites from both East and West Africa have a blend of small and large yield gaps. These potential improvements in crop production are also comparable with those from other yield gap studies. For example, in a global assessment Neumann et al. (2010) estimate that crop yields are between 50 and 64% of their maximum potential, which translates to potential yield improvements of between 56 and 100%. Neumann et al. (2010) used similar frontier-based methods as this study; however, their assessment is based on gridded spatial data which is likely to mask some of variability that would be present at the farm-level. In an assessment of yield gaps in African smallholder maize production across several countries, Tittonell and Giller (2013) estimated that observed yields on moderately fertile soils were between 36 and 61% of what could be attained under local condition, which suggests that yields could be increased by between 64 and 178%. Although Tittonell and Giller (2013) used a different approach, based on comparing average yields to maximum yields from field trials and top performing farmers, the findings are very similar to ours.

As mentioned, the nexus between productivity and environmental performance has recently attracted growing attention from the research community, with several studies including Burney et al., 2010, FAO, 2010 and Gerber et al. (2013) demonstrating the strong positive role that productivity improvements can play in lowering the emission intensity of agricultural production. This study adds further support to these findings, with sizeable reductions in emission intensities estimated for crop and livestock production. Emission intensities were estimated to fall by 20 to 63% for the main livestock products and by 25 to 62% for the main crop products, as a consequence of closing yield gaps. Closing yield gaps by improving technical efficiency will also generally improve the efficiency with which other natural resources, including land and water, are used. By changing from an output-oriented to an input-oriented frontier it would be possible to estimate absolute reductions in GHG emissions, and others in the use of resources such as land, for a given level of output. This presents a possible future extension of this study.

We should caution, however, that while we have controlled for variations in environmental factors on production that are beyond the control of farmers (e.g. precipitation and growing degree days) by confining each frontier model to geographically small sites, and by using a parametric approach that can account for statistical noise resulting from reporting errors, the estimated yield gaps may still incorporate some environmental factors beyond the control of farmers. Additionally, the standard assumption of TE scores from SFA and DEA frontier methods reflecting variations in farm performance under existing technologies and practices is not guaranteed. However, where the frontier sample populations are not in the process of rapid technological change this assumption can be upheld more easily. Since our study sites are characterized by low levels of technology adoption and innovation (owing to capital and scale constraints) and we focus on a single production year, we are confident that our results reflect the potential for improvement with existing practices. That said, it is impossible to rule out small differences in technologies across the sample just as it is impossible to exclude all factors beyond the control of farmers when estimating TE. For these reasons, our estimated potentials for improvements in yields and emission intensities must be viewed as upper bound estimates of what can be achieved without the introduction of new technologies and practices.

While the estimation of yield gaps provide useful benchmarks for policy makers about potential improvements, it is equally important to understand the drivers behind these gaps. To this end, our assessment of the link between farm attributes and yield gaps provides some possible site-specific leverage points, to help inform policy makers and extension agents in the design and targeting of capacity building programmes. We found a very strong and statistically significant link between market participation and farm performance in most sites, which suggests that efforts to promote market participation could be an important part of sustaining the closure yield gaps, particularly when farmers are able to produce in excess of their household needs. Further, three of the four most statistically significant relationships between market participation and efficiency were found in the most livestock orientated sites. While this reveals that smallholders tend to rely more on livestock production in areas with poor market access, it also indicates that measures to promote the participation in the market could be more beneficial in these areas.

The discernment of an efficiency dividend from the closer integration of crop and livestock enterprises was particularly instructive. There are a number of potentially beneficial synergies between livestock and crops which, while not explicitly analysed, can play an important role in raising the overall technical efficiency of the farm. The benefits of integration are derived from both the direct use of outputs from one enterprise into another and the use of by-products from one enterprise in another that would usually be left unexploited. For example, livestock can benefit crop production by providing organic fertilizer (manure) and traction. On the other hand, crops can benefit livestock production by providing feed in the form of residues. In this study, the benefits to integration were found to be larger for sites that were more specialized in either livestock or crop production. This finding is supported by the seminal work of McIntire, Bourzat, and Pingalii (1992), who showed that in more livestock dependent areas of Africa with low land productivity, crop production is not in competition with livestock and can provide residues for animal feed during times when pasture is less abundant.

There were also strong relationships between the age and gender of the farm household head and efficiency in some of the sites. In the few sites where these variables were significant they were associated with larger yield gaps. The link between farmer age and inefficiency is a relatively common finding Tipi et al., 2009, Battese and Coelli, 1995, Mathijs and Vranken, 2000, Bozoğlu and Ceyhan, 2007) and reflects the tendency for older farmers to be less innovative and receptive to extension initiatives than their younger counterparts. Similarly, female headed households tend to face larger yield gaps, with this relationship also being significantly in only a few sites. This may result from greater barriers to accessing input and output markets, and various farm services including extension together with higher risk aversion (Babu and Sanyal, 2014). As discussed, these findings could be used to help direct capacity building programmes to smallholders most in need of support, as well as indicate production structures that are most likely to perform efficiently. However, this assessment does not clearly discern which types of farms are likely to be the most receptive to technical support. While this study is an important first step, closer examination of and comparison of farms, including through field visits, would be needed to identify constraints and opportunities on a site-by-site basis.

Finally, it is important to note that there are a number of ways to estimate yield gaps. This study relies on the ex post measurement of performance gaps between farms assuming no change in existing practices and technologies. Another important approach is to estimate, ex ante, the potential for increasing productivity by adopting new technologies, including improved varieties and breeds of crops and livestock. These approaches involve different, but complementary ways to achieve similar goals.

## Figures and Tables

**Table 1:** Topographic, climatic and location characteristics of the sites.

Study site	Elevation (m above sea level)	Rainfall (mm/yr)	Distance to main city^a^ (km)	Main city's name — number of inhabitants
Nyando	1100–2500	900–1200	46	Kisumu — 259,258
Wote	900–1000	520	85	Machakos — 150,041
Hoima	620–1600	1400	36	Masindi — 94,622
Lushoto	900–2250	1200–1300	153	Tanga — 187,455
Borana	1000–2000	500–600	244	Arba Minch — 95,373
Yatenga	300–350	400–700	22.5	Ouahigouya — 73,153
Kaffrine	15–50	500–800	1	Kaffrine — 32, 942

*Source*: Förch et al. (2013) and SIPPEY (Système dInformation Populaire pour les Collectivités Locales au Sénégal) (2007).

a Distances to main city were calculated using Google Maps.

**Table 2:** Production characteristics of the study sites: a selection of the main farm inputs and outputs.

	Livestock (TLU index)	Labour (h)	Land (ha)	Farm assets (index)	Fertilizer (kg)	Milk (l)	Eggs (kg)	Grains (kg)	Legume–pulse (kg)	Vegetables (kg)	Fruit (kg)
*Nyando*											
Mean	6.8	981	4.3	12.9	2.5	988	209	1150	132	229	62
St. dev	5.0	934	4.6	8.0	15.1	1750	443	1261	337	664	337

*Wote*											
Mean	8.3	1421	4.5	11.5		182	108	403	288	15	1707
St. dev	6.1	1048	3.3	7.4		276	113	430	267	143	3928

*Hoima*											
Mean	3.8	2513	10.4	11.3	15.8	191	175	1112	395	1355	168
St. dev	5.9	1890	15.3	5.6	117.5	845	203	1592	670	1582	625

*Lushoto*											
Mean	2.2	2858	2.1	7.7	33.4	664	88	477	179	796	286
St. dev	2.2	2454	1.4	5.2	73.9	2276	158	491	209	2029	1696

*Borana*											
Mean	17.4	1633	3.7	11.1		1061	36	578	278		
St. dev	12.0	1595	2.6	6.6		1386	92	717	329		

*Yatenga*											
Mean	9.1	2332	4.6	14.5	65.0	77	28	1534	400	853	38
St. dev	12.0	4034	3.2	13.0	176.3	651	196	1374	543	1813	183

*Kaffrine*											
Mean	10.0	4474	26.3	12.8	333.1	133		2354	3015	246	1066
St. dev	9.8	3029	22.2	6.3	559.7	340		4286	10,080	647	2854

**Table 3:** The socio-economic attributes of farms and farmers in each study site.

	Age of household head (yrs)	Off farm income (%)	Household size (hd)	Market participation (%)	Domestic assets	Gender^a^ (% female head household)	Livestock specialization (%)
*Nyando*							
Mean	50.3	15.9	5.8	35.5	21.7	20.4	34.9
St. dev	14.0	14.1	2.2	26.8	31.5		23.3

*Wote*							
Mean	49.9	21.4	5.4	35.0	24.5	10.7	50.0
St. dev	13.1	41.9	2.0	21.3	41.2		23.6

*Hoima*							
Mean	46.1	43.5	7.0	54.7	33.9	11.6	20.4
St. dev	13.6	126.3	2.7	23.7	35.6		22.5

*Lushoto*							
Mean	51.3	20.5	4.8	39.3	9.2	27.6	19.5
St. dev	45.4	45.4	1.7	25.5	10.6		25.0

*Borana*							
Mean	46.4	10.0	6.4	17.0	3.5	14.3	59.2
St. dev	15.2	26.7	2.4	21.9	3.0		24.6

*Yatenga*							
Mean	50.3	78.6	10.6	31.4	64.3	4.7	33.3
St. dev	14.1	268.4	4.7	78.0	49.0		27.0

*Kaffrine*							
Mean	53.0	27.4	12.4	38.4	29.5	1.0	24.8
St. dev	13.4	39.7	3.7	24.7	20.1		26.4

a The gender variable is modelled as a dummy variable, but for expository purposes it is displayed here as the percentage of female headed households in each site.

**Table 4:** The average technical efficiency scores and yield gap estimates for each site.

	Nyando	Wote	Hoima	Lushoto	Borana	Yatenga	Kaffrine
Mean TE	0.56	0.70	0.63	0.46	0.43	0.57	0.72
Yield gap (%)	79	43	58	115	133	76	39
CV (%)	49	30	28	57	49	43	25

**Table 5:** The potential percentage increases in the production of the main livestock and crop products as a consequence of closing yield gaps.

	Milk	Eggs	Chicken	Grains	Beans/pulses	Tubers/roots
Nyando	55%	96%	98%	77%	49%	58%
Wote	40%	37%	33%	39%	36%	28%
Hoima	28%	56%	47%	46%	65%	70%
Lushoto	45%	154%		155%	136%	209%
Borana	167%	102%		97%	108%	
Yatenga	100%	38%	127%	68%	75%	48%
Kaffrine	38%		67%	33%	33%	16%

**Table 6:** The relationship between technical inefficiency and socio-economic farm attributes, including coefficient values and levels of significance for each variable.

	Nyando	Wote	Hoima	Lushoto	Borana	Yatenga	Kaffrine
Age	0.025^a^	0.004	0.023^b^		0.0001	− 0.063	− 0.001
Off farm income	0.003^c^	0.008^a^	0.0005	0.006	0.009^a^	0.002^c^	
Household size	0.040	0.067			0.029	0.315^d^	
Market participat.	− 0.035^a^	− 0.029^b^	− 0.029^d^	− 0.019^c^	− 0.007 ^c^	− 0.058	− 0.060
Domestic assets	− 0.005	− 0.027	0.007	− 0.022	− 0.027	− 0.029^d^	
Gender	0.38^c^	− 0.054	− 0.18	1.16^a^	0.378^c^	− 4.61	− 3.67
Livestock special.	0.000	0.010^c^	− 0.017	− 0.037^d^	0.014^b^	0.023^d^	− 0.071

a, b, c, and d indicate the level of statistical significance: a (0.001); b (0.01); c (0.05); d (0.1).

**Table 7:** Hypothesis test; null hypothesis specifies that inefficiency effects are absent from the model.

	Test statistic (z-value)
Nyando	2.45^c^
Wote	2.67^c^
Hoima	2.07^c^
Lushoto	1.67^d^
Borana	1.65^d^
Yatenga	2.98^b^
Kaffrine	2.15 ^c^

a, b, c, and d indicate the level of statistical significance: a (0.001); b (0.01); c (0.05); d (0.1).

**Table 8:** Hypothesis test; null hypothesis specifies that inefficiency effects are not a function of the farm attribute variables.

	Log (likelihood)	Test statistic (Chi-sq)
Nyando	− 144.2	101.3^a^
Wote	− 80.1	60.0^a^
Hoima	− 148.2	23.9^a^
Lushoto	− 156.4	31.6^a^
Borana	− 127.6	32.4^a^
Yatenga	− 133.1	22.7^b^
Kaffrine	− 58.5	13.37^b^

a, b, c, and d indicate the level of statistical significance: a (0.001); b (0.01); c (0.05); d (0.1).

**Table 9:** Changes in the emission intensity (EI) of livestock products (kg CO_2_eq/kg protein) from closing yield gaps.

	Baseline EI of poultry products	Efficient EI of poultry products	% reduct.	Baseline EI of ruminant products	Efficient EI of ruminant products	% reduct.
Nyando	0.3	0.1	49%	250	161	36%
Wote	0.8	0.3	27%	868	615	29%
Hoima	0.6	0.4	36%	475	371	22%
Lushoto	0.6	0.2	61%	209	141	33%
Borana	0.7	0.4	50%	615	230	63%
Yatenga	2.0	1.3	34%	563	298	47%
Kaffrine	4.5	2.7	40%	271	216	20%

**Table 10:** Changes in the emission intensity (EI) of selected crop products (kg CO_2_eq/MJ) from closing yield gaps.

	Baseline EI of grains	Efficient EI of grains	% reduct.	Baseline EI of beans/pulses	Efficient EI of beans/pulses	% reduct.
Nyando	0.003	0.002	43%	0.007	0.005	33%
Wote	0.011	0.008	28%	0.029	0.021	27%
Hoima	0.006	0.004	32%	0.005	0.003	39%
Lushoto	0.012	0.005	61%	0.007	0.003	58%
Borana	0.003	0.002	58%	0.006	0.002	62%
Yatenga	0.009	0.005	40%	0.006	0.003	43%
Kaffrine	0.019	0.014	25%	0.006	0.005	25%

## Appendix A Supplementary data

Supplementary material for Closing system-wide yield gaps to increase food production and mitigate GHGs among mixed crop-livestock smallholders in Sub-Saharan Africa
