# Supplementary material for: Closing system-wide yield gaps to increase food production and mitigate GHGs among mixed crop–livestock smallholders in Sub-Saharan Africa
Source: Agric Syst. 2016 Mar;143:106–13. doi: 10.1016/j.agsy.2015.12.006 (PMC4767044; doi:10.1016/j.agsy.2015.12.006)
Supplement: Supplementary file 1 — Supplementary material for Closing system-wide yield gaps to increase food production and mitigate GHGs among mixed crop-livestock smallholders in Sub-Saharan Africa [file mmc1.docx]

**Supplementary material for Closing system-wide yield gaps to increase food production and mitigate GHGs among mixed crop-livestock smallholders in Sub-Saharan Africa**

Henderson B^a*^, Godde C^a^, Medina-Hidalgo D^a^, van Wijk M^b^, Silvestri S^b^, Douxchamps S^b^, Stephenson E^a^, Power B^a^, Rigolot C^a^, Cacho O^c^ Herrero M^a^

1. Commonwealth Scientific and Industrial Research Organization, Queensland Bioscience Precinct, 306 Carmody Road, St Lucia, Qld, 4067, Australia
2. ILRI, International Livestock Research Institute, Nairobi, Kenya
3. University of New England, Armidale, NSW, 2351

* Corresponding author e-mail: [ben.henderson@csiro.au](mailto:ben.henderson@csiro.au)

**Supplementary material description, data and results**

The means and standard deviations of the input and output variables used in the stochastic frontier models for each site, are listed in table S1. For animals, tropical livestock unit (TLU) index was used to aggregate different animal types. This index takes into account the feed requirements of different animals and are therefore reflective of their varying resource requirements (ILRI, 2011; FAO, 2003). The standard measure for one TLU is one cattle with a body weight of 250 kg. By contrast a 30 kg sheep or goat with is equal to 0.2 TLUs, and is therefore assumed to consume 20 percent as much feed as 250 kg cow. For farm assets we relied on ILRI (2011) and BMFG (2010) to aggregate different asset classes. These included all productive farm assets including items such as ploughs, water pumps and wheelbarrows. The values assigned to each type of asset reflect their relative economic values. For example, a powered water pump has a value twelve times greater than a shovel and three times greater than a plough (ILRI, 2011; BMFG 2010). For livestock products, crop products and materials (which includes fertilizers, seeds, pesticides, herbicides, feeds, vaccinations) we constructed Fisher quantity indexes (Diewert 1992). This required the use of quantity and price data from IMPACTlite database (Rufino et al. 2013).

**Table S1** Summary of the production inputs and outputs used in the stochastic frontier models

| **Site** | **Livestock**  **(TLU index)** | **Labour**  **(hrs)** | **Land**  **(ha)** | **Farm assets**  **(Index)** | **Materials**  **(Fisher quantity index)** | **Livestock**  **(Fisher quantity index)** | **Crops**  **(Fisher quantity index)** |
| --- | --- | --- | --- | --- | --- | --- | --- |
| **Nyando** |  |  |  |  |  |  |  |
| mean | 6.8 | 981 | 4.3 | 12.9 | 0.28 | 0.75 | 0.26 |
| st. dev | 5.0 | 934 | 4.6 | 8.0 | 1.39 | 1.22 | 0.36 |
| **Wote** |  |  |  |  |  |  |  |
| mean | 8.3 | 1,421 | 4.5 | 11.5 | 0.19 | 0.41 | 0.36 |
| st. dev | 6.1 | 1,048 | 3.3 | 7.4 | 0.38 | 0.32 | 0.35 |
| **Hoima** |  |  |  |  |  |  |  |
| mean | 3.8 | 2,513 | 10.4 | 11.3 | 1.65 | 0.06 | 2.25 |
| st. dev | 5.9 | 1,890 | 15.3 | 5.6 | 4.97 | 0.14 | 4.13 |
| **Lushoto** |  |  |  |  |  |  |  |
| mean | 2.2 | 2,858 | 2.1 | 7.7 | 1.05 | 0.23 | 0.96 |
| st. dev | 2.2 | 2,454 | 1.4 | 5.2 | 2.64 | 0.71 | 3.25 |
| **Borana** |  |  |  |  |  |  |  |
| mean | 17.4 | 1,633 | 3.7 | 11.1 | 90.88 | 47.50 | 5.29 |
| st. dev | 12.0 | 1,595 | 2.6 | 6.6 | 243.81 | 119.00 | 5.40 |
| **Yatenga** |  |  |  |  |  |  |  |
| mean | 9.1 | 2,332 | 4.6 | 14.5 | 3.45 | 6.07 | 0.71 |
| st. dev | 12.0 | 4,034 | 3.2 | 13.0 | 13.20 | 12.38 | 0.99 |
| **Kaffrine** |  |  |  |  |  |  |  |
| mean | 10.0 | 4,474 | 26.3 | 12.8 | 0.58 | 0.23 | 0.97 |
| st. dev | 9.8 | 3,029 | 22.2 | 6.3 | 0.63 | 0.34 | 1.04 |

The parameter definitions listed below in Table S2 correspond to the production function estimates for each site shown in Tables S3-9. The farm attribute variables (Off farm income, Gender, Household size, Market participation, Livestock specialisation, Domestic assets) that were included as explanatory variables for the technical inefficiency estimates are listed at the bottom of Tables S3-9.

**Table S2** Parameter definitions

| **Parameter** | **Definition** |
| --- | --- |
| a_0 | Intercept |
| a_1 | Land |
| a_2 | Labour |
| a_3 | Livestock |
| a_4 | Materials |
| a_5 | Farm_assets |
| a_6 | Crops/Livestock |
| b_1_1 | Land^2^ |
| b_1_2 | Land x Labour |
| b_1_3 | Land x Livestock |
| b_1_4 | Land x Materials |
| b_1_5 | Land x Farm_assets |
| b_1_6 | Land x Crops/Livestock |
| b_2_2 | Labour^2^ |
| b_2_3 | Labour x Livestock |
| b_2_4 | Labour x Materials |
| b_2_5 | Labour x Farm_assets |
| b_2_6 | Labour x Crops/Livestock |
| b_3_3 | Livestock^2^ |
| b_3_4 | Livestock x Materials |
| b_3_5 | Livestock x Farm_assets |
| b_3_6 | Livestock x Crops/Livestock |
| b_4_4 | Materials^2^ |
| b_4_5 | Materials x Farm_assets |
| b_4_6 | Materials x Crops/Livestock |
| b_5_5 | Farm_assets^2^ |
| b_6_6 | Crops/Livestock ^2^ |

Tables S3-9 below contain the complete list of parameter estimates of the stochastic frontier models for each study site. For each variable the tables include coefficient estimate values, standard errors as well as tests of statistical significance including Z scores and P-values, with the associated levels of statistical significance indicated in the final column of each table.

**Table S3** Maximum likelihood estimates of the translog stochastic production frontier model for Nyando

| **Parameter** | **Estimate** | **Std. Error** | **z value** | **Pr(>\|z\|)** |  |
| --- | --- | --- | --- | --- | --- |
| a_0 | 0.173 | 0.245 | 0.71 | 0.479 |  |
| a_1 | 0.699 | 0.249 | 2.80 | 0.005 | ** |
| a_2 | 0.377 | 0.239 | 1.58 | 0.114 |  |
| a_3 | -0.029 | 0.206 | -0.14 | 0.888 |  |
| a_4 | -0.133 | 0.085 | -1.56 | 0.118 |  |
| a_5 | -0.399 | 0.264 | -1.51 | 0.131 |  |
| a_6 | -0.774 | 0.129 | -5.99 | 0.000 | *** |
| b_1_1 | 0.264 | 0.155 | 1.70 | 0.089 | . |
| b_1_2 | -0.062 | 0.106 | -0.59 | 0.554 |  |
| b_1_3 | -0.278 | 0.108 | -2.57 | 0.010 | * |
| b_1_4 | 0.026 | 0.040 | 0.66 | 0.511 |  |
| b_1_5 | -0.106 | 0.138 | -0.77 | 0.442 |  |
| b_1_6 | -0.074 | 0.066 | -1.13 | 0.258 |  |
| b_2_2 | 0.202 | 0.134 | 1.50 | 0.133 |  |
| b_2_3 | 0.008 | 0.104 | 0.07 | 0.942 |  |
| b_2_4 | 0.007 | 0.049 | 0.14 | 0.890 |  |
| b_2_5 | -0.079 | 0.124 | -0.63 | 0.526 |  |
| b_2_6 | 0.052 | 0.059 | 0.87 | 0.386 |  |
| b_3_3 | 0.031 | 0.088 | 0.35 | 0.730 |  |
| b_3_4 | 0.068 | 0.054 | 1.27 | 0.205 |  |
| b_3_5 | -0.031 | 0.102 | -0.30 | 0.762 |  |
| b_3_6 | 0.030 | 0.042 | 0.73 | 0.468 |  |
| b_4_4 | -0.049 | 0.026 | -1.93 | 0.054 | . |
| b_4_5 | -0.078 | 0.060 | -1.30 | 0.193 |  |
| b_4_6 | -0.019 | 0.025 | -0.76 | 0.447 |  |
| b_5_5 | 0.200 | 0.212 | 0.95 | 0.344 |  |
| b_5_6 | 0.062 | 0.066 | 0.94 | 0.347 |  |
| b_6_6 | -0.110 | 0.035 | -3.14 | 0.002 | ** |
| Age | 0.025 | 0.005 | 5.34 | 0.000 | *** |
| Off farm income | 0.003 | 0.001 | 2.44 | 0.015 | * |
| Gender | 0.382 | 0.220 | 1.73 | 0.083 | . |
| Household size | 0.040 | 0.037 | 1.10 | 0.271 |  |
| Market participation | -0.035 | 0.008 | -4.62 | 0.000 | *** |
| Livestock specialisation | 0.000 | 0.008 | 0.00 | 0.996 |  |
| Domestic assets | -0.005 | 0.004 | -1.27 | 0.205 |  |
| sigmaSq | 0.446 | 0.121 | 3.70 | 0.000 | *** |
| lambda | 1.366 | 0.557 | 2.45 | 0.014 | * |
| log likelihood function | -144.198 |  |  |  |  |

“***”, “**”, “*”, “.” indicate level of statistical significance: “***” (0.001); “**” (0.01); “*” (0.05); “.” (0.1)

**Table S4** Maximum likelihood estimates of the translog stochastic production frontier model for Lushoto

| **Parameter** | **Estimate** | **Std. Error** | **z value** | **Pr(>\|z\|)** |  |
| --- | --- | --- | --- | --- | --- |
| a_0 | -0.071 | 0.247 | -0.287 | 0.774 |  |
| a_1 | 0.536 | 0.258 | 2.080 | 0.038 | * |
| a_2 | 0.531 | 0.248 | 2.138 | 0.033 | * |
| a_3 | -0.081 | 0.211 | -0.383 | 0.702 |  |
| a_4 | 0.293 | 0.161 | 1.826 | 0.068 | . |
| a_5 | 0.340 | 0.296 | 1.149 | 0.251 |  |
| a_6 | -0.575 | 0.094 | -6.102 | 0.000 | *** |
| b_1_1 | 0.110 | 0.285 | 0.384 | 0.701 |  |
| b_1_2 | -0.064 | 0.196 | -0.324 | 0.746 |  |
| b_1_3 | 0.556 | 0.205 | 2.715 | 0.007 | ** |
| b_1_4 | 0.101 | 0.102 | 0.988 | 0.323 |  |
| b_1_5 | -0.225 | 0.188 | -1.196 | 0.232 |  |
| b_1_6 | 0.187 | 0.061 | 3.084 | 0.002 | ** |
| b_2_2 | -0.327 | 0.183 | -1.780 | 0.075 | . |
| b_2_3 | -0.326 | 0.146 | -2.236 | 0.025 | * |
| b_2_4 | 0.243 | 0.098 | 2.477 | 0.013 | * |
| b_2_5 | -0.084 | 0.181 | -0.464 | 0.642 |  |
| b_2_6 | -0.115 | 0.057 | -2.028 | 0.043 | * |
| b_3_3 | -0.069 | 0.220 | -0.315 | 0.753 |  |
| b_3_4 | 0.219 | 0.110 | 1.989 | 0.047 | * |
| b_3_5 | 0.405 | 0.165 | 2.452 | 0.014 | * |
| b_3_6 | 0.016 | 0.052 | 0.299 | 0.765 |  |
| b_4_4 | -0.152 | 0.078 | -1.939 | 0.053 | . |
| b_4_5 | -0.161 | 0.132 | -1.218 | 0.223 |  |
| b_4_6 | 0.029 | 0.035 | 0.833 | 0.405 |  |
| b_5_5 | -0.723 | 0.262 | -2.765 | 0.006 | ** |
| b_5_6 | 0.027 | 0.070 | 0.393 | 0.694 |  |
| b_6_6 | -0.058 | 0.018 | -3.134 | 0.002 | ** |
| Livestock specialisation | -0.037 | 0.021 | -1.746 | 0.081 | . |
| Market participation | -0.019 | 0.008 | -2.509 | 0.012 | * |
| Gender | 1.158 | 0.317 | 3.650 | 0.000 | *** |
| Domestic assets | -0.023 | 0.024 | -0.945 | 0.345 |  |
| Household size | 0.264 | 0.054 | 4.898 | 0.000 | *** |
| Off farm income | 0.006 | 0.004 | 1.573 | 0.116 |  |
| sigmaSq | 1.218 | 0.351 | 3.475 | 0.001 | *** |
| lambda | 3.081 | 1.827 | 1.686 | 0.092 | . |
| log likelihood function | -156.364 |  |  |  |  |

“***”, “**”, “*”, “.” indicate level of statistical significance: “***” (0.001); “**” (0.01); “*” (0.05); “.” (0.1)

**Table S5** Maximum likelihood estimates of the translog stochastic production frontier model for Wote

| **Parameter** | **Estimate** | **Std. Error** | **z value** | **Pr(>\|z\|)** |  |
| --- | --- | --- | --- | --- | --- |
| a_0 | -0.431 | 0.175 | -2.456 | 0.014 | * |
| a_1 | 0.053 | 0.137 | 0.391 | 0.696 |  |
| a_2 | -0.048 | 0.139 | -0.345 | 0.730 |  |
| a_3 | 0.151 | 0.133 | 1.137 | 0.256 |  |
| a_4 | -0.062 | 0.062 | -1.007 | 0.314 |  |
| a_5 | 0.434 | 0.216 | 2.009 | 0.045 | * |
| a_6 | -0.443 | 0.089 | -4.994 | 0.000 | *** |
| b_1_1 | 0.219 | 0.146 | 1.500 | 0.134 |  |
| b_1_2 | 0.069 | 0.118 | 0.586 | 0.558 |  |
| b_1_3 | -0.057 | 0.064 | -0.892 | 0.373 |  |
| b_1_4 | 0.002 | 0.029 | 0.056 | 0.955 |  |
| b_1_5 | -0.175 | 0.136 | -1.290 | 0.197 |  |
| b_1_6 | 0.126 | 0.058 | 2.153 | 0.031 | * |
| b_2_2 | -0.039 | 0.146 | -0.269 | 0.788 |  |
| b_2_3 | 0.019 | 0.072 | 0.259 | 0.796 |  |
| b_2_4 | -0.039 | 0.035 | -1.110 | 0.267 |  |
| b_2_5 | 0.018 | 0.110 | 0.168 | 0.866 |  |
| b_2_6 | -0.100 | 0.054 | -1.857 | 0.063 | . |
| b_3_3 | -0.023 | 0.082 | -0.274 | 0.784 |  |
| b_3_4 | 0.033 | 0.024 | 1.362 | 0.173 |  |
| b_3_5 | -0.080 | 0.096 | -0.833 | 0.405 |  |
| b_3_6 | 0.089 | 0.042 | 2.120 | 0.034 | * |
| b_4_4 | -0.020 | 0.010 | -1.982 | 0.047 | * |
| b_4_5 | 0.002 | 0.040 | 0.052 | 0.958 |  |
| b_4_6 | -0.005 | 0.017 | -0.319 | 0.750 |  |
| b_5_5 | 0.368 | 0.207 | 1.783 | 0.075 | . |
| b_5_6 | -0.111 | 0.060 | -1.859 | 0.063 | . |
| b_6_6 | -0.124 | 0.032 | -3.820 | 0.000 | *** |
| Age | 0.004 | 0.006 | 0.608 | 0.543 |  |
| Gender | -0.055 | 0.332 | -0.164 | 0.870 |  |
| Household size | 0.067 | 0.051 | 1.317 | 0.188 |  |
| Market participation | -0.029 | 0.010 | -2.782 | 0.005 | ** |
| Domestic assets | -0.027 | 0.018 | -1.475 | 0.140 |  |
| Off farm income | 0.008 | 0.002 | 3.380 | 0.001 | *** |
| Livestock specialisation | 0.010 | 0.005 | 1.973 | 0.049 | * |
| sigmaSq | 0.342 | 0.115 | 2.961 | 0.003 | ** |
| lambda | 1.604 | 0.601 | 2.671 | 0.008 | ** |
| log likelihood function | -80.150 |  |  |  |  |

“***”, “**”, “*”, “.” indicate level of statistical significance: “***” (0.001); “**” (0.01); “*” (0.05); “.” (0.1)

**Table S6** Maximum likelihood estimates of the translog stochastic production frontier model for Hoima

| **Parameter** | **Estimate** | **Std. Error** | **z value** | **Pr(>\|z\|)** |  |
| --- | --- | --- | --- | --- | --- |
| a_0 | -0.592 | 0.323 | -1.835 | 0.067 | . |
| a_1 | 0.196 | 0.222 | 0.882 | 0.378 |  |
| a_3 | 0.414 | 0.251 | 1.652 | 0.099 | . |
| a_4 | -0.081 | 0.099 | -0.815 | 0.415 |  |
| a_5 | 1.205 | 0.452 | 2.669 | 0.008 | ** |
| a_6 | -0.401 | 0.088 | -4.539 | 0.000 | *** |
| b_1_1 | -0.186 | 0.109 | -1.708 | 0.088 | . |
| b_1_3 | 0.181 | 0.112 | 1.618 | 0.106 |  |
| b_1_4 | -0.012 | 0.045 | -0.257 | 0.797 |  |
| b_1_5 | -0.255 | 0.189 | -1.350 | 0.177 |  |
| b_1_6 | 0.124 | 0.055 | 2.253 | 0.024 | * |
| b_3_3 | 0.115 | 0.127 | 0.906 | 0.365 |  |
| b_3_4 | -0.064 | 0.045 | -1.426 | 0.154 |  |
| b_3_5 | -0.075 | 0.164 | -0.458 | 0.647 |  |
| b_3_6 | -0.052 | 0.042 | -1.259 | 0.208 |  |
| b_4_4 | 0.029 | 0.033 | 0.868 | 0.385 |  |
| b_4_5 | -0.051 | 0.102 | -0.502 | 0.616 |  |
| b_4_6 | 0.018 | 0.026 | 0.707 | 0.480 |  |
| b_5_5 | 1.114 | 0.459 | 2.426 | 0.015 | * |
| b_5_6 | -0.133 | 0.085 | -1.565 | 0.118 |  |
| b_6_6 | -0.107 | 0.022 | -4.892 | 0.000 | *** |
| Market participation | -0.029 | 0.016 | -1.804 | 0.071 | . |
| Off farm income | 0.000 | 0.001 | 0.529 | 0.597 |  |
| Age | 0.023 | 0.008 | 3.017 | 0.003 | ** |
| Domestic assets | 0.007 | 0.005 | 1.313 | 0.189 |  |
| Livestock specialisation | -0.017 | 0.016 | -1.036 | 0.300 |  |
| Gender | -0.179 | 0.605 | -0.296 | 0.767 |  |
| sigmaSq | 0.985 | 0.500 | 1.970 | 0.049 | * |
| lambda | 1.630 | 0.787 | 2.071 | 0.038 | * |
| log likelihood function | -148.188 |  |  |  |  |

“***”, “**”, “*”, “.” indicate level of statistical significance: “***” (0.001); “**” (0.01); “*” (0.05); “.” (0.1)

**Table S7** Maximum likelihood estimates of the translog stochastic production frontier model for Borana

| **Parameter** | **Estimate** | **Std. Error** | **z value** | **Pr(>\|z\|)** |  |
| --- | --- | --- | --- | --- | --- |
| a_0 | -0.606 | 0.456 | -1.328 | 0.184 |  |
| a_1 | 0.184 | 0.283 | 0.649 | 0.516 |  |
| a_2 | -0.064 | 0.455 | -0.141 | 0.888 |  |
| a_3 | 0.863 | 0.310 | 2.786 | 0.005 | ** |
| a_4 | -0.146 | 0.174 | -0.837 | 0.402 |  |
| a_5 | 1.390 | 0.782 | 1.777 | 0.076 | . |
| a_6 | -0.533 | 0.130 | -4.097 | 0.000 | *** |
| b_1_1 | -0.173 | 0.130 | -1.325 | 0.185 |  |
| b_1_2 | 0.169 | 0.125 | 1.353 | 0.176 |  |
| b_1_3 | 0.217 | 0.118 | 1.847 | 0.065 | . |
| b_1_4 | -0.027 | 0.051 | -0.524 | 0.600 |  |
| b_1_5 | -0.385 | 0.238 | -1.620 | 0.105 |  |
| b_1_6 | 0.161 | 0.067 | 2.420 | 0.016 | * |
| b_2_2 | -0.314 | 0.248 | -1.264 | 0.206 |  |
| b_2_3 | 0.307 | 0.125 | 2.452 | 0.014 | * |
| b_2_4 | -0.098 | 0.070 | -1.407 | 0.159 |  |
| b_2_5 | 0.244 | 0.351 | 0.695 | 0.487 |  |
| b_2_6 | 0.065 | 0.077 | 0.846 | 0.397 |  |
| b_3_3 | 0.040 | 0.130 | 0.307 | 0.759 |  |
| b_3_4 | -0.089 | 0.043 | -2.061 | 0.039 | * |
| b_3_5 | -0.207 | 0.192 | -1.078 | 0.281 |  |
| b_3_6 | -0.120 | 0.046 | -2.622 | 0.009 | ** |
| b_4_4 | 0.049 | 0.035 | 1.412 | 0.158 |  |
| b_4_5 | 0.009 | 0.102 | 0.090 | 0.928 |  |
| b_4_6 | 0.003 | 0.029 | 0.100 | 0.921 |  |
| b_5_5 | 1.288 | 0.620 | 2.077 | 0.038 | * |
| b_5_6 | -0.107 | 0.098 | -1.096 | 0.273 |  |
| b_6_6 | -0.037 | 0.023 | -1.567 | 0.117 |  |
| Age | 0.042 | 0.021 | 2.010 | 0.044 | * |
| Off farm income | 0.005 | 0.004 | 1.455 | 0.146 |  |
| Livestock specialisation | -0.545 | 0.311 | -1.753 | 0.080 | . |
| Market participation | -0.031 | 0.015 | -2.073 | 0.038 | * |
| Gender | -0.136 | 0.993 | -0.137 | 0.891 |  |
| Household size | 0.078 | 0.097 | 0.810 | 0.418 |  |
| Domestic assets | 0.018 | 0.008 | 2.312 | 0.021 | * |
| sigmaSq | 1.316 | 0.591 | 2.227 | 0.026 | * |
| lambda | 1.793 | 0.607 | 2.955 | 0.003 | ** |
| log likelihood function | -143.811 |  |  |  |  |

“***”, “**”, “*”, “.” indicate level of statistical significance: “***” (0.001); “**” (0.01); “*” (0.05); “.” (0.1)

**Table S8** Maximum likelihood estimates of the translog stochastic production frontier model for Yatenga

| **Parameter** | **Estimate** | **Std. Error** | **z value** | **Pr(>\|z\|)** |  |
| --- | --- | --- | --- | --- | --- |
| a_0 | 0.673 | 0.152 | 4.434 | 0.000 | *** |
| a_1 | 0.499 | 0.148 | 3.381 | 0.001 | *** |
| a_3 | -0.087 | 0.130 | -0.666 | 0.505 |  |
| a_4 | -0.121 | 0.065 | -1.874 | 0.061 | . |
| a_5 | 0.217 | 0.176 | 1.231 | 0.219 |  |
| a_6 | -0.656 | 0.068 | -9.666 | 0.000 | *** |
| b_1_1 | -0.297 | 0.187 | -1.590 | 0.112 |  |
| b_1_3 | 0.073 | 0.089 | 0.828 | 0.408 |  |
| b_1_4 | -0.010 | 0.072 | -0.139 | 0.889 |  |
| b_1_5 | -0.022 | 0.157 | -0.142 | 0.887 |  |
| b_1_6 | 0.047 | 0.057 | 0.821 | 0.411 |  |
| b_3_3 | 0.011 | 0.112 | 0.096 | 0.923 |  |
| b_3_4 | -0.078 | 0.044 | -1.781 | 0.075 | . |
| b_3_5 | 0.026 | 0.136 | 0.191 | 0.848 |  |
| b_3_6 | -0.081 | 0.062 | -1.312 | 0.189 |  |
| b_4_4 | 0.009 | 0.034 | 0.277 | 0.782 |  |
| b_4_5 | -0.086 | 0.065 | -1.328 | 0.184 |  |
| b_4_6 | -0.040 | 0.031 | -1.299 | 0.194 |  |
| b_5_5 | -0.230 | 0.116 | -1.984 | 0.047 | * |
| b_5_6 | 0.109 | 0.064 | 1.688 | 0.091 | . |
| b_6_6 | 0.021 | 0.031 | 0.659 | 0.510 |  |
| Off farm income | 0.002 | 0.001 | 2.439 | 0.015 | * |
| Household size | 0.315 | 0.171 | 1.845 | 0.065 | . |
| Market participation | -0.058 | 0.040 | -1.453 | 0.146 |  |
| Domestic assets | -0.029 | 0.017 | -1.698 | 0.090 | . |
| Livestock specialisation | 0.023 | 0.011 | 2.036 | 0.042 | * |
| Gender | -4.609 | 5.730 | -0.804 | 0.421 |  |
| Age | -0.063 | 0.050 | -1.252 | 0.211 |  |
| sigmaSq | 2.491 | 1.287 | 1.936 | 0.053 | . |
| lambda | 4.115 | 1.379 | 2.983 | 0.003 | ** |
| log likelihood function | -133.100 |  |  |  |  |

“***”, “**”, “*”, “.” indicate level of statistical significance: “***” (0.001); “**” (0.01); “*” (0.05); “.” (0.1)

**Table S9** Maximum likelihood estimates of the translog stochastic production frontier model for Kaffrine

| **Parameter** | **Estimate** | **Std. Error** | **z value** | **Pr(>\|z\|)** |  |
| --- | --- | --- | --- | --- | --- |
| a_0 | 0.354 | 0.245 | 1.442 | 0.149 |  |
| a_1 | -0.004 | 0.330 | -0.012 | 0.991 |  |
| a_2 | 0.363 | 0.354 | 1.026 | 0.305 |  |
| a_3 | 0.474 | 0.180 | 2.635 | 0.008 | ** |
| a_4 | 0.291 | 0.187 | 1.552 | 0.121 |  |
| a_5 | 0.111 | 0.299 | 0.373 | 0.710 |  |
| a_6 | -0.836 | 0.128 | -6.513 | 0.000 | *** |
| b_1_1 | -0.694 | 0.289 | -2.406 | 0.016 | * |
| b_1_2 | 0.511 | 0.177 | 2.884 | 0.004 | ** |
| b_1_3 | -0.265 | 0.173 | -1.535 | 0.125 |  |
| b_1_4 | 0.340 | 0.175 | 1.939 | 0.053 | . |
| b_1_5 | 0.562 | 0.268 | 2.099 | 0.036 | * |
| b_1_6 | -0.018 | 0.065 | -0.278 | 0.781 |  |
| b_2_2 | -0.814 | 0.289 | -2.817 | 0.005 | ** |
| b_2_3 | 0.154 | 0.147 | 1.051 | 0.293 |  |
| b_2_4 | 0.138 | 0.155 | 0.890 | 0.373 |  |
| b_2_5 | -0.033 | 0.225 | -0.146 | 0.884 |  |
| b_2_6 | -0.090 | 0.055 | -1.630 | 0.103 |  |
| b_3_3 | 0.103 | 0.107 | 0.964 | 0.335 |  |
| b_3_4 | 0.063 | 0.083 | 0.759 | 0.448 |  |
| b_3_5 | -0.238 | 0.137 | -1.736 | 0.082 | . |
| b_3_6 | 0.027 | 0.042 | 0.642 | 0.521 |  |
| b_4_4 | -0.273 | 0.101 | -2.691 | 0.007 | ** |
| b_4_5 | -0.168 | 0.117 | -1.442 | 0.149 |  |
| b_4_6 | -0.015 | 0.038 | -0.401 | 0.689 |  |
| b_5_5 | 0.062 | 0.243 | 0.255 | 0.798 |  |
| b_5_6 | -0.040 | 0.057 | -0.694 | 0.488 |  |
| b_6_6 | -0.042 | 0.031 | -1.356 | 0.175 |  |
| Market participation | -0.060 | 0.072 | -0.835 | 0.404 |  |
| Livestock specialisation | -0.071 | 0.095 | -0.749 | 0.454 |  |
| Gender | -3.679 | 23.940 | -0.154 | 0.878 |  |
| Age | -0.001 | 0.026 | -0.050 | 0.960 |  |
| sigmaSq | 1.744 | 1.712 | 1.019 | 0.308 |  |
| lambda | 4.537 | 2.107 | 2.153 | 0.031 | * |
| log likelihood function | -58.481 |  |  |  |  |

“***”, “**”, “*”, “.” indicate level of statistical significance: “***” (0.001); “**” (0.01); “*” (0.05); “.” (0.1)

**References**

BMGF (Bill and Melinda Gates Foundation). (2010) *Agricultural Development Outcome Indicators: Initiative and Sub-Initiative Progress Indicators & Pyramid of Outcome Indicators*. BMGF, Seattle.

Diewert, W. E. (1992) Gisher Ideal Output, Input and Productivity Indexes Revisited. *Journal of Productivity Analysis* 3: 211–48.

FAO (2003). *Compendium of Agricultural – Environmental Indicators*. Statistics Division, FAO, Rome.

Rufino, M. C., Quiros, C., Boureima, M., Desta, S., Douxchamps, S., Herrero, M., Kiplimo, J., Lamissa, D., Mango, J., Moussa, A. S., Naab, J., Ndour, Y., Sayula, G., Silvestri, S., Singh, D., Teufel, N., Wanyaman, I. (2013) *Developing Generic Tools for Characterizing Agricultural Systems for Climate and Global Change Studies (IMPACTlite – Phase 2)*. ILRI to the CGIAR Research Program on Climate Change, Agriculture and Food Security (CCAFS).
